# A new face of orexins action - neuroprotection

**DOI:** 10.1186/2193-1801-4-S1-L59

**Published:** 2015-06-12

**Authors:** Jolanta Zawilska

**Affiliations:** Department of Pharmacodynamics, Medical University of Lodz, Poland

**Keywords:** orexins, neuroprotection, oxidate stress

Orexins A and B (hypocretins 1 and 2) are two closely related peptides produced mainly by hypothalamic neurons that project to numerous brain structures. Orexins exert their biological activity by binding to two subtypes of GPCR receptors, OX1R and OX2R. The orexin system has been shown to orchestrate multifaceted physiological functions, including vigilance and the sleep/wake cycle, energy homeostasis, endocrine, visceral functions and pathological states, such as narcolepsy and drug abuse [1]. In addition, a neuroprotective potential of orexin A has been recently demonstrated in rats using a model of focal cerebral ischemia [2]. In our studies we investigated effects of orexins on survival of cultured neurons from the rat cerebral cortex. Quantitative real-time PCR revealed the presence of OX1R and OX2R in cortical neurons. Orexins and [Ala11-D-Leu15]orexin B (a selective agonist of OX2R), used at 0.001-1 microM concentrations, markedly increased neuronal cells viability, an effect associated with an attenuation of caspase-3 activity. Comparable potency of the three peptides suggests a predominant role of OX2R in the studied phenomenon. Under conditions of chemical hypoxia, orexins potently increased neuronal viability and protected cortical neurons from oxidative stress. The pro-survival effect of orexins was blocked by U0126 and 10-DEBC, inhibitors of MEKK and Akt, respectively. In addition, orexins A and B stimulated Akt and ERK1/2 in cortical neurons in a time- and concentration-dependent manner. It is suggested that both Akt and ERK1/2 play an important role in the pro-survival effects of orexins in neurons.

## References

[1] Li J, Hu Z, de Lecea L (2014). Br J Pharmacol.

[2] Kitamura E, Hamada J, Kanazawa N, Yonekura J, Masuda R, Sakai F, Mochizuki H (2010). Neurosci Res.

